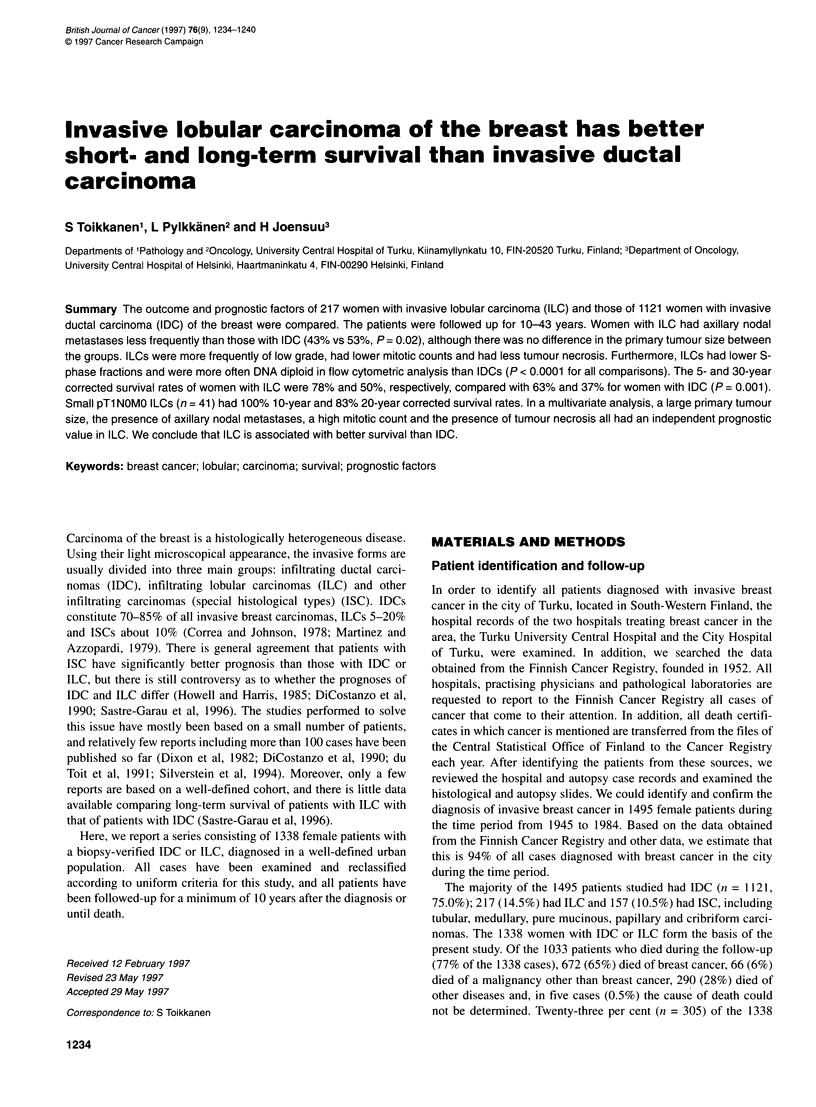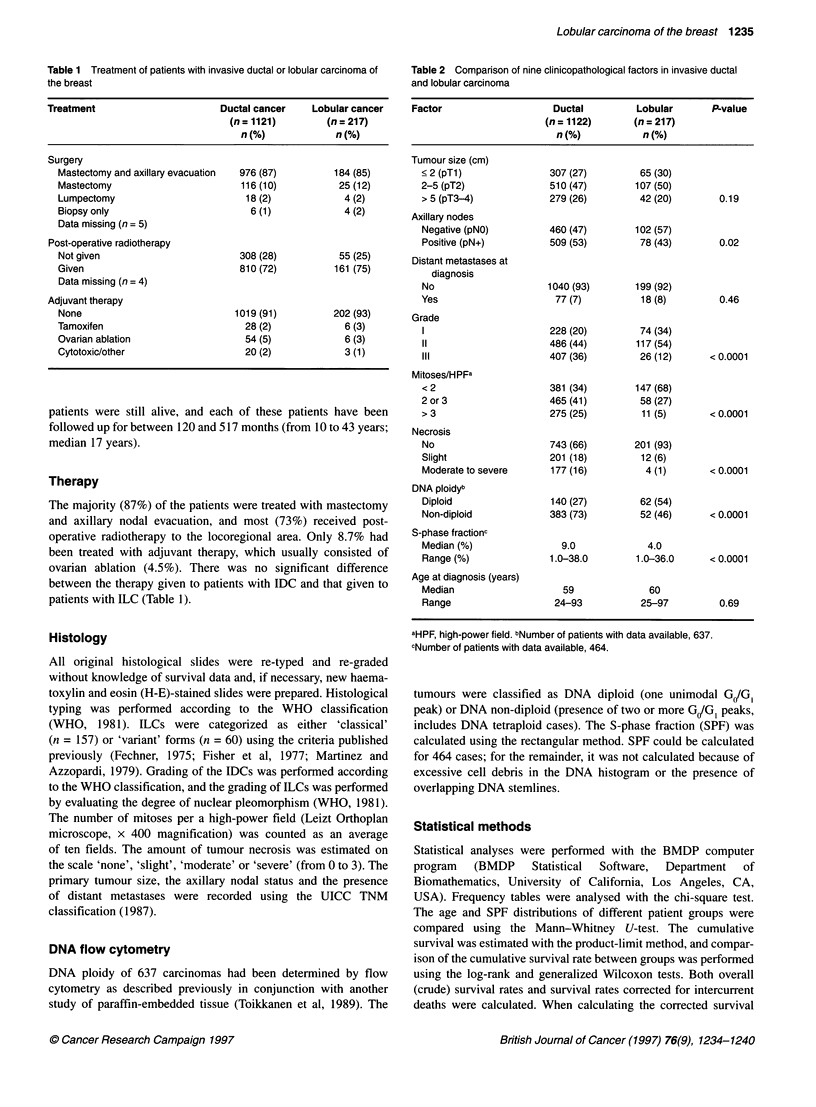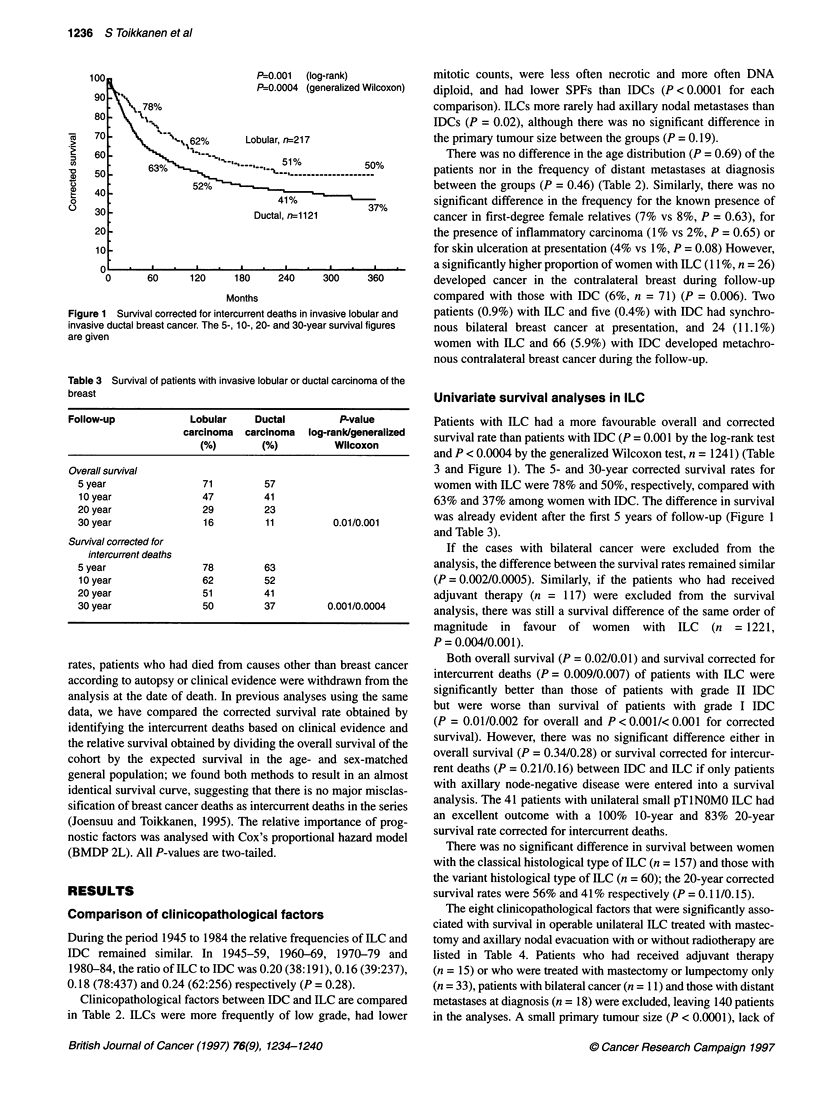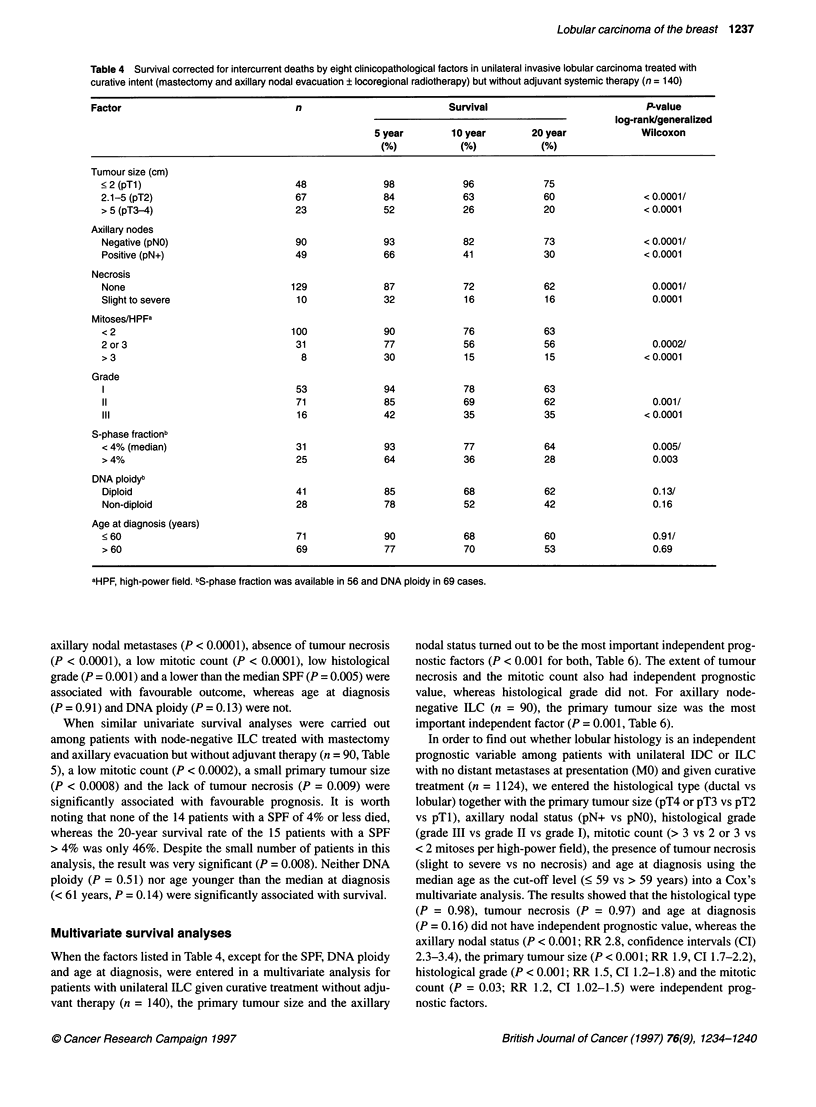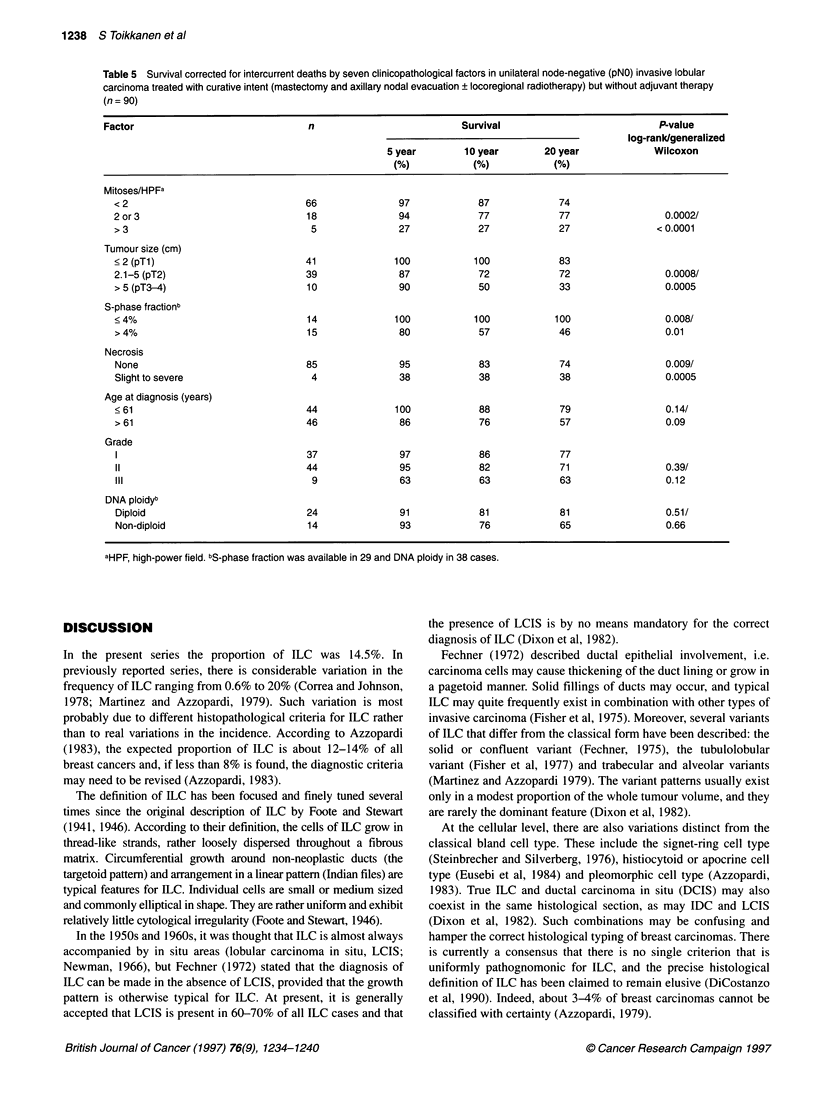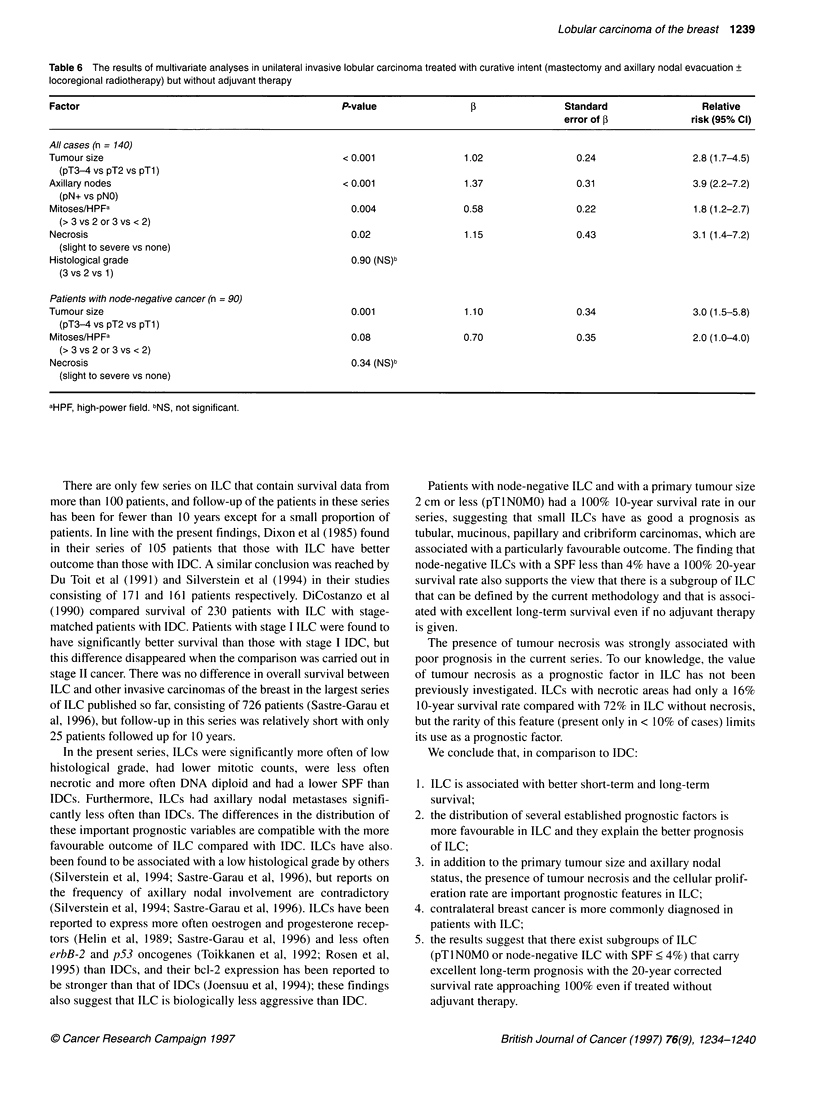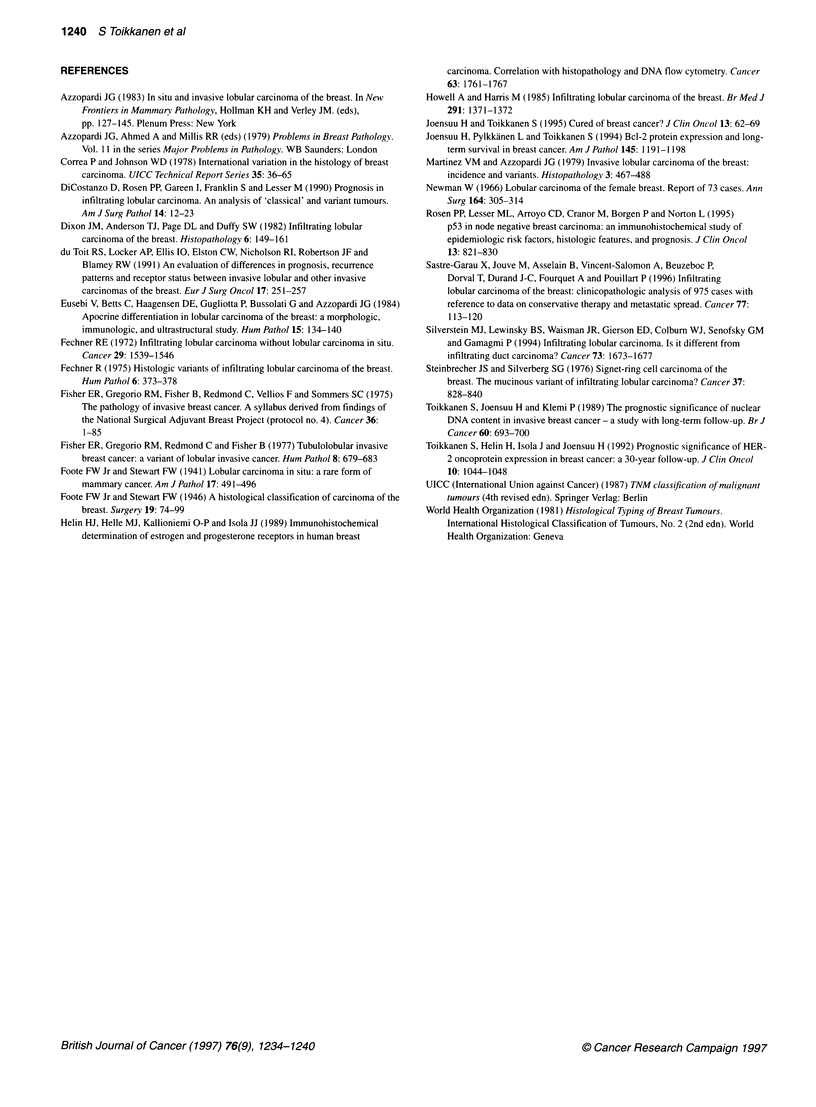# Invasive lobular carcinoma of the breast has better short- and long-term survival than invasive ductal carcinoma.

**DOI:** 10.1038/bjc.1997.540

**Published:** 1997

**Authors:** S. Toikkanen, L. PylkkÃ¤nen, H. Joensuu

**Affiliations:** Department of Pathology, University Central Hospital of Turku, Finland.

## Abstract

The outcome and prognostic factors of 217 women with invasive lobular carcinoma (ILC) and those of 1121 women with invasive ductal carcinoma (IDC) of the breast were compared. The patients were followed up for 10-43 years. Women with ILC had axillary nodal metastases less frequently than those with IDC (43% vs 53%, P = 0.02), although there was no difference in the primary tumour size between the groups. ILCs were more frequently of low grade, had lower mitotic counts and had less tumour necrosis. Furthermore, ILCs had lower S-phase fractions and were more often DNA diploid in flow cytometric analysis than IDCs (P < 0.0001 for all comparisons). The 5- and 30-year corrected survival rates of women with ILC were 78% and 50%, respectively, compared with 63% and 37% for women with IDC (P = 0.001). Small pT1NOMO ILCs (n = 41) had 100% 10-year and 83% 20-year corrected survival rates. In a multivariate analysis, a large primary tumour size, the presence of axillary nodal metastases, a high mitotic count and the presence of tumour necrosis all had an independent prognostic value in ILC. We conclude that ILC is associated with better survival than IDC.


British Journal of Cancer (1997) 76(9), 1234–1240
© 1997 Cancer Research Campaign

### Invasive lobular carcinoma of the breast has better short- and long-term survival than invasive ductal carcinoma

**S Toikkanen[1], L Pylkkänen[2] and H Joensuu[3]**

Departments of [1]Pathology and [2]Oncology, University Central Hospital of Turku, Kiinamyllynkatu 10, FIN-20520 Turku, Finland; [3]Department of Oncology, University Central Hospital of Helsinki, Haartmaninkatu 4, FIN-00290 Helsinki, Finland

**Summary** The outcome and prognostic factors of 217 women with invasive lobular carcinoma (ILC) and those of 1121 women with invasive ductal carcinoma (IDC) of the breast were compared. The patients were followed up for 10–43 years. Women with ILC had axillary nodal metastases less frequently than those with IDC (43% vs 53%, $P = 0.02$), although there was no difference in the primary tumour size between the groups. ILCs were more frequently of low grade, had lower mitotic counts and had less tumour necrosis. Furthermore, ILCs had lower S-phase fractions and were more often DNA diploid in flow cytometric analysis than IDCs ($P < 0.0001$ for all comparisons). The 5- and 30-year corrected survival rates of women with ILC were 78% and 50%, respectively, compared with 63% and 37% for women with IDC ($P = 0.001$). Small pT1N0M0 ILCs ($n = 41$) had 100% 10-year and 83% 20-year corrected survival rates. In a multivariate analysis, a large primary tumour size, the presence of axillary nodal metastases, a high mitotic count and the presence of tumour necrosis all had an independent prognostic value in ILC. We conclude that ILC is associated with better survival than IDC.

**Keywords:** breast cancer; lobular; carcinoma; survival; prognostic factors

Carcinoma of the breast is a histologically heterogeneous disease. Using their light microscopical appearance, the invasive forms are usually divided into three main groups: infiltrating ductal carcinomas (IDC), infiltrating lobular carcinomas (ILC) and other infiltrating carcinomas (special histological types) (ISC). IDCs constitute 70–85% of all invasive breast carcinomas, ILCs 5–20% and ISCs about 10% (Correa and Johnson, 1978; Martinez and Azzopardi, 1979). There is general agreement that patients with ISC have significantly better prognosis than those with IDC or ILC, but there is still controversy as to whether the prognoses of IDC and ILC differ (Howell and Harris, 1985; DiCostanzo et al, 1990; Sastre-Garau et al, 1996). The studies performed to solve this issue have mostly been based on a small number of patients, and relatively few reports including more than 100 cases have been published so far (Dixon et al, 1982; DiCostanzo et al, 1990; du Toit et al, 1991; Silverstein et al, 1994). Moreover, only a few reports are based on a well-defined cohort, and there is little data available comparing long-term survival of patients with ILC with that of patients with IDC (Sastre-Garau et al, 1996).

Here, we report a series consisting of 1338 female patients with a biopsy-verified IDC or ILC, diagnosed in a well-defined urban population. All cases have been examined and reclassified according to uniform criteria for this study, and all patients have been followed-up for a minimum of 10 years after the diagnosis or until death.

*Received 12 February 1997*
*Revised 23 May 1997*
*Accepted 29 May 1997*

*Correspondence to:* S Toikkanen

### MATERIALS AND METHODS

### Patient identification and follow-up

In order to identify all patients diagnosed with invasive breast cancer in the city of Turku, located in South-Western Finland, the hospital records of the two hospitals treating breast cancer in the area, the Turku University Central Hospital and the City Hospital of Turku, were examined. In addition, we searched the data obtained from the Finnish Cancer Registry, founded in 1952. All hospitals, practising physicians and pathological laboratories are requested to report to the Finnish Cancer Registry all cases of cancer that come to their attention. In addition, all death certificates in which cancer is mentioned are transferred from the files of the Central Statistical Office of Finland to the Cancer Registry each year. After identifying the patients from these sources, we reviewed the hospital and autopsy case records and examined the histological and autopsy slides. We could identify and confirm the diagnosis of invasive breast cancer in 1495 female patients during the time period from 1945 to 1984. Based on the data obtained from the Finnish Cancer Registry and other data, we estimate that this is 94% of all cases diagnosed with breast cancer in the city during the time period.

The majority of the 1495 patients studied had IDC ($n$ = 1121, 75.0%); 217 (14.5%) had ILC and 157 (10.5%) had ISC, including tubular, medullary, pure mucinous, papillary and cribriform carcinomas. The 1338 women with IDC or ILC form the basis of the present study. Of the 1033 patients who died during the follow-up (77% of the 1338 cases), 672 (65%) died of breast cancer, 66 (6%) died of a malignancy other than breast cancer, 290 (28%) died of other diseases and, in five cases (0.5%) the cause of death could not be determined. Twenty-three per cent ($n$ = 305) of the 1338

**Table 1** Treatment of patients with invasive ductal or lobular carcinoma of the breast

| Treatment | Ductal cancer ($n$ = 1121) $n$ (%) | Lobular cancer ($n$ = 217) $n$ (%) |
|---|---|---|
| Surgery | | |
| Mastectomy and axillary evacuation | 976 (87) | 184 (85) |
| Mastectomy | 116 (10) | 25 (12) |
| Lumpectomy | 18 (2) | 4 (2) |
| Biopsy only | 6 (1) | 4 (2) |
| Data missing ($n$ = 5) | | |
| Post-operative radiotherapy | | |
| Not given | 308 (28) | 55 (25) |
| Given | 810 (72) | 161 (75) |
| Data missing ($n$ = 4) | | |
| Adjuvant therapy | | |
| None | 1019 (91) | 202 (93) |
| Tamoxifen | 28 (2) | 6 (3) |
| Ovarian ablation | 54 (5) | 6 (3) |
| Cytotoxic/other | 20 (2) | 3 (1) |

patients were still alive, and each of these patients have been followed up for between 120 and 517 months (from 10 to 43 years; median 17 years).

### Therapy

The majority (87%) of the patients were treated with mastectomy and axillary nodal evacuation, and most (73%) received post-operative radiotherapy to the locoregional area. Only 8.7% had been treated with adjuvant therapy, which usually consisted of ovarian ablation (4.5%). There was no significant difference between the therapy given to patients with IDC and that given to patients with ILC (Table 1).

### Histology

All original histological slides were re-typed and re-graded without knowledge of survival data and, if necessary, new haematoxylin and eosin (H-E)-stained slides were prepared. Histological typing was performed according to the WHO classification (WHO, 1981). ILCs were categorized as either 'classical' ($n$ = 157) or 'variant' forms ($n$ = 60) using the criteria published previously (Fechner, 1975; Fisher et al, 1977; Martinez and Azzopardi, 1979). Grading of the IDCs was performed according to the WHO classification, and the grading of ILCs was performed by evaluating the degree of nuclear pleomorphism (WHO, 1981). The number of mitoses per a high-power field (Leizt Orthoplan microscope, × 400 magnification) was counted as an average of ten fields. The amount of tumour necrosis was estimated on the scale 'none', 'slight', 'moderate' or 'severe' (from 0 to 3). The primary tumour size, the axillary nodal status and the presence of distant metastases were recorded using the UICC TNM classification (1987).

### DNA flow cytometry

DNA ploidy of 637 carcinomas had been determined by flow cytometry as described previously in conjunction with another study of paraffin-embedded tissue (Toikkanen et al, 1989). The tumours were classified as DNA diploid (one unimodal $G_0/G_1$ peak) or DNA non-diploid (presence of two or more $G_0/G_1$ peaks, includes DNA tetraploid cases). The S-phase fraction (SPF) was calculated using the rectangular method. SPF could be calculated for 464 cases; for the remainder, it was not calculated because of excessive cell debris in the DNA histogram or the presence of overlapping DNA stemlines.

**Table 2** Comparison of nine clinicopathological factors in invasive ductal and lobular carcinoma

| Factor | Ductal ($n$ = 1122) $n$ (%) | Lobular ($n$ = 217) $n$ (%) | $P$-value |
|---|---|---|---|
| Tumour size (cm) | | | |
| ≤ 2 (pT1) | 307 (27) | 65 (30) | |
| 2–5 (pT2) | 510 (47) | 107 (50) | |
| > 5 (pT3–4) | 279 (26) | 42 (20) | 0.19 |
| Axillary nodes | | | |
| Negative (pN0) | 460 (47) | 102 (57) | |
| Positive (pN+) | 509 (53) | 78 (43) | 0.02 |
| Distant metastases at diagnosis | | | |
| No | 1040 (93) | 199 (92) | |
| Yes | 77 (7) | 18 (8) | 0.46 |
| Grade | | | |
| I | 228 (20) | 74 (34) | |
| II | 486 (44) | 117 (54) | |
| III | 407 (36) | 26 (12) | < 0.0001 |
| Mitoses/HPF[a] | | | |
| < 2 | 381 (34) | 147 (68) | |
| 2 or 3 | 465 (41) | 58 (27) | |
| > 3 | 275 (25) | 11 (5) | < 0.0001 |
| Necrosis | | | |
| No | 743 (66) | 201 (93) | |
| Slight | 201 (18) | 12 (6) | |
| Moderate to severe | 177 (16) | 4 (1) | < 0.0001 |
| DNA ploidy[b] | | | |
| Diploid | 140 (27) | 62 (54) | |
| Non-diploid | 383 (73) | 52 (46) | < 0.0001 |
| S-phase fraction[c] | | | |
| Median (%) | 9.0 | 4.0 | |
| Range (%) | 1.0–38.0 | 1.0–36.0 | < 0.0001 |
| Age at diagnosis (years) | | | |
| Median | 59 | 60 | |
| Range | 24–93 | 25–97 | 0.69 |

[a]HPF, high-power field. [b]Number of patients with data available, 637. [c]Number of patients with data available, 464.

### Statistical methods

Statistical analyses were performed with the BMDP computer program (BMDP Statistical Software, Department of Biomathematics, University of California, Los Angeles, CA, USA). Frequency tables were analysed with the chi-square test. The age and SPF distributions of different patient groups were compared using the Mann–Whitney $U$-test. The cumulative survival was estimated with the product-limit method, and comparison of the cumulative survival rate between groups was performed using the log-rank and generalized Wilcoxon tests. Both overall (crude) survival rates and survival rates corrected for intercurrent deaths were calculated. When calculating the corrected survival

© Cancer Research Campaign 1997

**Figure 1** Survival corrected for intercurrent deaths in invasive lobular and invasive ductal breast cancer. The 5-, 10-, 20- and 30-year survival figures are given

**Table 3** Survival of patients with invasive lobular or ductal carcinoma of the breast

| Follow-up | Lobular carcinoma (%) | Ductal carcinoma (%) | *P*-value log-rank/generalized Wilcoxon |
|---|---|---|---|
| *Overall survival* | | | |
| 5 year | 71 | 57 | |
| 10 year | 47 | 41 | |
| 20 year | 29 | 23 | |
| 30 year | 16 | 11 | 0.01/0.001 |
| *Survival corrected for intercurrent deaths* | | | |
| 5 year | 78 | 63 | |
| 10 year | 62 | 52 | |
| 20 year | 51 | 41 | |
| 30 year | 50 | 37 | 0.001/0.0004 |

rates, patients who had died from causes other than breast cancer according to autopsy or clinical evidence were withdrawn from the analysis at the date of death. In previous analyses using the same data, we have compared the corrected survival rate obtained by identifying the intercurrent deaths based on clinical evidence and the relative survival obtained by dividing the overall survival of the cohort by the expected survival in the age- and sex-matched general population; we found both methods to result in an almost identical survival curve, suggesting that there is no major misclassification of breast cancer deaths as intercurrent deaths in the series (Joensuu and Toikkanen, 1995). The relative importance of prognostic factors was analysed with Cox's proportional hazard model (BMDP 2L). All *P*-values are two-tailed.

### RESULTS

### Comparison of clinicopathological factors

During the period 1945 to 1984 the relative frequencies of ILC and IDC remained similar. In 1945–59, 1960–69, 1970–79 and 1980–84, the ratio of ILC to IDC was 0.20 (38:191), 0.16 (39:237), 0.18 (78:437) and 0.24 (62:256) respectively ($P$ = 0.28).

Clinicopathological factors between IDC and ILC are compared in Table 2. ILCs were more frequently of low grade, had lower mitotic counts, were less often necrotic and more often DNA diploid, and had lower SPFs than IDCs ($P < 0.0001$ for each comparison). ILCs more rarely had axillary nodal metastases than IDCs ($P$ = 0.02), although there was no significant difference in the primary tumour size between the groups ($P$ = 0.19).

There was no difference in the age distribution ($P$ = 0.69) of the patients nor in the frequency of distant metastases at diagnosis between the groups ($P$ = 0.46) (Table 2). Similarly, there was no significant difference in the frequency for the known presence of cancer in first-degree female relatives (7% vs 8%, $P$ = 0.63), for the presence of inflammatory carcinoma (1% vs 2%, $P$ = 0.65) or for skin ulceration at presentation (4% vs 1%, $P$ = 0.08) However, a significantly higher proportion of women with ILC (11%, $n$ = 26) developed cancer in the contralateral breast during follow-up compared with those with IDC (6%, $n$ = 71) ($P$ = 0.006). Two patients (0.9%) with ILC and five (0.4%) with IDC had synchronous bilateral breast cancer at presentation, and 24 (11.1%) women with ILC and 66 (5.9%) with IDC developed metachronous contralateral breast cancer during the follow-up.

### Univariate survival analyses in ILC

Patients with ILC had a more favourable overall and corrected survival rate than patients with IDC ($P$ = 0.001 by the log-rank test and $P < 0.0004$ by the generalized Wilcoxon test, $n$ = 1241) (Table 3 and Figure 1). The 5- and 30-year corrected survival rates for women with ILC were 78% and 50%, respectively, compared with 63% and 37% among women with IDC. The difference in survival was already evident after the first 5 years of follow-up (Figure 1 and Table 3).

If the cases with bilateral cancer were excluded from the analysis, the difference between the survival rates remained similar ($P$ = 0.002/0.0005). Similarly, if the patients who had received adjuvant therapy ($n$ = 117) were excluded from the survival analysis, there was still a survival difference of the same order of magnitude in favour of women with ILC ($n$ = 1221, $P$ = 0.004/0.001).

Both overall survival ($P$ = 0.02/0.01) and survival corrected for intercurrent deaths ($P$ = 0.009/0.007) of patients with ILC were significantly better than those of patients with grade II IDC but were worse than survival of patients with grade I IDC ($P$ = 0.01/0.002 for overall and $P < 0.001/< 0.001$ for corrected survival). However, there was no significant difference either in overall survival ($P$ = 0.34/0.28) or survival corrected for intercurrent deaths ($P$ = 0.21/0.16) between IDC and ILC if only patients with axillary node-negative disease were entered into a survival analysis. The 41 patients with unilateral small pT1N0M0 ILC had an excellent outcome with a 100% 10-year and 83% 20-year survival rate corrected for intercurrent deaths.

There was no significant difference in survival between women with the classical histological type of ILC ($n$ = 157) and those with the variant histological type of ILC ($n$ = 60); the 20-year corrected survival rates were 56% and 41% respectively ($P$ = 0.11/0.15).

The eight clinicopathological factors that were significantly associated with survival in operable unilateral ILC treated with mastectomy and axillary nodal evacuation with or without radiotherapy are listed in Table 4. Patients who had received adjuvant therapy ($n$ = 15) or who were treated with mastectomy or lumpectomy only ($n$ = 33), patients with bilateral cancer ($n$ = 11) and those with distant metastases at diagnosis ($n$ = 18) were excluded, leaving 140 patients in the analyses. A small primary tumour size ($P$ < 0.0001), lack of

© Cancer Research Campaign 1997

**Table 4** Survival corrected for intercurrent deaths by eight clinicopathological factors in unilateral invasive lobular carcinoma treated with curative intent (mastectomy and axillary nodal evacuation ± locoregional radiotherapy) but without adjuvant systemic therapy ($n$ = 140)

| Factor | *n* | Survival | | | *P*-value log-rank/generalized Wilcoxon |
|---|---|---|---|---|---|
| | | 5 year (%) | 10 year (%) | 20 year (%) | |
| Tumour size (cm) | | | | | |
| ≤ 2 (pT1) | 48 | 98 | 96 | 75 | |
| 2.1–5 (pT2) | 67 | 84 | 63 | 60 | < 0.0001/ |
| > 5 (pT3–4) | 23 | 52 | 26 | 20 | < 0.0001 |
| Axillary nodes | | | | | |
| Negative (pN0) | 90 | 93 | 82 | 73 | < 0.0001/ |
| Positive (pN+) | 49 | 66 | 41 | 30 | < 0.0001 |
| Necrosis | | | | | |
| None | 129 | 87 | 72 | 62 | 0.0001/ |
| Slight to severe | 10 | 32 | 16 | 16 | 0.0001 |
| Mitoses/HPF[a] | | | | | |
| < 2 | 100 | 90 | 76 | 63 | |
| 2 or 3 | 31 | 77 | 56 | 56 | 0.0002/ |
| > 3 | 8 | 30 | 15 | 15 | < 0.0001 |
| Grade | | | | | |
| I | 53 | 94 | 78 | 63 | |
| II | 71 | 85 | 69 | 62 | 0.001/ |
| III | 16 | 42 | 35 | 35 | < 0.0001 |
| S-phase fraction[b] | | | | | |
| < 4% (median) | 31 | 93 | 77 | 64 | 0.005/ |
| > 4% | 25 | 64 | 36 | 28 | 0.003 |
| DNA ploidy[b] | | | | | |
| Diploid | 41 | 85 | 68 | 62 | 0.13/ |
| Non-diploid | 28 | 78 | 52 | 42 | 0.16 |
| Age at diagnosis (years) | | | | | |
| ≤ 60 | 71 | 90 | 68 | 60 | 0.91/ |
| > 60 | 69 | 77 | 70 | 53 | 0.69 |

[a]HPF, high-power field. [b]S-phase fraction was available in 56 and DNA ploidy in 69 cases.

axillary nodal metastases ($P$ < 0.0001), absence of tumour necrosis ($P$ < 0.0001), a low mitotic count ($P$ < 0.0001), low histological grade ($P$ = 0.001) and a lower than the median SPF ($P$ = 0.005) were associated with favourable outcome, whereas age at diagnosis ($P$ = 0.91) and DNA ploidy ($P$ = 0.13) were not.

When similar univariate survival analyses were carried out among patients with node-negative ILC treated with mastectomy and axillary evacuation but without adjuvant therapy ($n$ = 90, Table 5), a low mitotic count ($P$ < 0.0002), a small primary tumour size ($P$ < 0.0008) and the lack of tumour necrosis ($P$ = 0.009) were significantly associated with favourable prognosis. It is worth noting that none of the 14 patients with a SPF of 4% or less died, whereas the 20-year survival rate of the 15 patients with a SPF > 4% was only 46%. Despite the small number of patients in this analysis, the result was very significant ($P$ = 0.008). Neither DNA ploidy ($P$ = 0.51) nor age younger than the median at diagnosis (< 61 years, $P$ = 0.14) were significantly associated with survival.

### Multivariate survival analyses

When the factors listed in Table 4, except for the SPF, DNA ploidy and age at diagnosis, were entered in a multivariate analysis for patients with unilateral ILC given curative treatment without adjuvant therapy ($n$ = 140), the primary tumour size and the axillary nodal status turned out to be the most important independent prognostic factors ($P$ < 0.001 for both, Table 6). The extent of tumour necrosis and the mitotic count also had independent prognostic value, whereas histological grade did not. For axillary node-negative ILC ($n$ = 90), the primary tumour size was the most important independent factor ($P$ = 0.001, Table 6).

In order to find out whether lobular histology is an independent prognostic variable among patients with unilateral IDC or ILC with no distant metastases at presentation (M0) and given curative treatment ($n$ = 1124), we entered the histological type (ductal vs lobular) together with the primary tumour size (pT4 or pT3 vs pT2 vs pT1), axillary nodal status (pN+ vs pN0), histological grade (grade III vs grade II vs grade I), mitotic count (> 3 vs 2 or 3 vs < 2 mitoses per high-power field), the presence of tumour necrosis (slight to severe vs no necrosis) and age at diagnosis using the median age as the cut-off level (≤ 59 vs > 59 years) into a Cox's multivariate analysis. The results showed that the histological type ($P$ = 0.98), tumour necrosis ($P$ = 0.97) and age at diagnosis ($P$ = 0.16) did not have independent prognostic value, whereas the axillary nodal status ($P$ < 0.001; RR 2.8, confidence intervals (CI) 2.3–3.4), the primary tumour size ($P$ < 0.001; RR 1.9, CI 1.7–2.2), histological grade ($P$ < 0.001; RR 1.5, CI 1.2–1.8) and the mitotic count ($P$ = 0.03; RR 1.2, CI 1.02–1.5) were independent prognostic factors.

© Cancer Research Campaign 1997

**Table 5** Survival corrected for intercurrent deaths by seven clinicopathological factors in unilateral node-negative (pN0) invasive lobular carcinoma treated with curative intent (mastectomy and axillary nodal evacuation ± locoregional radiotherapy) but without adjuvant therapy ($n$ = 90)

| Factor | $n$ | Survival | | | $P$-value log-rank/generalized Wilcoxon |
|---|---|---|---|---|---|
| | | 5 year (%) | 10 year (%) | 20 year (%) | |
| Mitoses/HPF[a] | | | | | |
| < 2 | 66 | 97 | 87 | 74 | |
| 2 or 3 | 18 | 94 | 77 | 77 | 0.0002/ |
| > 3 | 5 | 27 | 27 | 27 | < 0.0001 |
| Tumour size (cm) | | | | | |
| ≤ 2 (pT1) | 41 | 100 | 100 | 83 | |
| 2.1–5 (pT2) | 39 | 87 | 72 | 72 | 0.0008/ |
| > 5 (pT3–4) | 10 | 90 | 50 | 33 | 0.0005 |
| S-phase fraction[b] | | | | | |
| ≤ 4% | 14 | 100 | 100 | 100 | 0.008/ |
| > 4% | 15 | 80 | 57 | 46 | 0.01 |
| Necrosis | | | | | |
| None | 85 | 95 | 83 | 74 | 0.009/ |
| Slight to severe | 4 | 38 | 38 | 38 | 0.0005 |
| Age at diagnosis (years) | | | | | |
| ≤ 61 | 44 | 100 | 88 | 79 | 0.14/ |
| > 61 | 46 | 86 | 76 | 57 | 0.09 |
| Grade | | | | | |
| I | 37 | 97 | 86 | 77 | |
| II | 44 | 95 | 82 | 71 | 0.39/ |
| III | 9 | 63 | 63 | 63 | 0.12 |
| DNA ploidy[b] | | | | | |
| Diploid | 24 | 91 | 81 | 81 | 0.51/ |
| Non-diploid | 14 | 93 | 76 | 65 | 0.66 |

[a]HPF, high-power field. [b]S-phase fraction was available in 29 and DNA ploidy in 38 cases.

### DISCUSSION

In the present series the proportion of ILC was 14.5%. In previously reported series, there is considerable variation in the frequency of ILC ranging from 0.6% to 20% (Correa and Johnson, 1978; Martinez and Azzopardi, 1979). Such variation is most probably due to different histopathological criteria for ILC rather than to real variations in the incidence. According to Azzopardi (1983), the expected proportion of ILC is about 12–14% of all breast cancers and, if less than 8% is found, the diagnostic criteria may need to be revised (Azzopardi, 1983).

The definition of ILC has been focused and finely tuned several times since the original description of ILC by Foote and Stewart (1941, 1946). According to their definition, the cells of ILC grow in thread-like strands, rather loosely dispersed throughout a fibrous matrix. Circumferential growth around non-neoplastic ducts (the targetoid pattern) and arrangement in a linear pattern (Indian files) are typical features for ILC. Individual cells are small or medium sized and commonly elliptical in shape. They are rather uniform and exhibit relatively little cytological irregularity (Foote and Stewart, 1946).

In the 1950s and 1960s, it was thought that ILC is almost always accompanied by in situ areas (lobular carcinoma in situ, LCIS; Newman, 1966), but Fechner (1972) stated that the diagnosis of ILC can be made in the absence of LCIS, provided that the growth pattern is otherwise typical for ILC. At present, it is generally accepted that LCIS is present in 60–70% of all ILC cases and that the presence of LCIS is by no means mandatory for the correct diagnosis of ILC (Dixon et al, 1982).

Fechner (1972) described ductal epithelial involvement, i.e. carcinoma cells may cause thickening of the duct lining or grow in a pagetoid manner. Solid fillings of ducts may occur, and typical ILC may quite frequently exist in combination with other types of invasive carcinoma (Fisher et al, 1975). Moreover, several variants of ILC that differ from the classical form have been described: the solid or confluent variant (Fechner, 1975), the tubulolobular variant (Fisher et al, 1977) and trabecular and alveolar variants (Martinez and Azzopardi 1979). The variant patterns usually exist only in a modest proportion of the whole tumour volume, and they are rarely the dominant feature (Dixon et al, 1982).

At the cellular level, there are also variations distinct from the classical bland cell type. These include the signet-ring cell type (Steinbrecher and Silverberg, 1976), histiocytoid or apocrine cell type (Eusebi et al, 1984) and pleomorphic cell type (Azzopardi, 1983). True ILC and ductal carcinoma in situ (DCIS) may also coexist in the same histological section, as may IDC and LCIS (Dixon et al, 1982). Such combinations may be confusing and hamper the correct histological typing of breast carcinomas. There is currently a consensus that there is no single criterion that is uniformly pathognomonic for ILC, and the precise histological definition of ILC has been claimed to remain elusive (DiCostanzo et al, 1990). Indeed, about 3–4% of breast carcinomas cannot be classified with certainty (Azzopardi, 1979).

*© Cancer Research Campaign 1997*

**Table 6** The results of multivariate analyses in unilateral invasive lobular carcinoma treated with curative intent (mastectomy and axillary nodal evacuation ± locoregional radiotherapy) but without adjuvant therapy

| Factor | *P*-value | β | Standard error of β | Relative risk (95% CI) |
|---|---|---|---|---|
| *All cases* (*n* = *140*) | | | | |
| Tumour size (pT3–4 vs pT2 vs pT1) | < 0.001 | 1.02 | 0.24 | 2.8 (1.7–4.5) |
| Axillary nodes (pN+ vs pN0) | < 0.001 | 1.37 | 0.31 | 3.9 (2.2–7.2) |
| Mitoses/HPF[a] (> 3 vs 2 or 3 vs < 2) | 0.004 | 0.58 | 0.22 | 1.8 (1.2–2.7) |
| Necrosis (slight to severe vs none) | 0.02 | 1.15 | 0.43 | 3.1 (1.4–7.2) |
| Histological grade (3 vs 2 vs 1) | 0.90 (NS)[b] | | | |
| *Patients with node-negative cancer* (*n* = *90*) | | | | |
| Tumour size (pT3–4 vs pT2 vs pT1) | 0.001 | 1.10 | 0.34 | 3.0 (1.5–5.8) |
| Mitoses/HPF[a] (> 3 vs 2 or 3 vs < 2) | 0.08 | 0.70 | 0.35 | 2.0 (1.0–4.0) |
| Necrosis (slight to severe vs none) | 0.34 (NS)[b] | | | |

[a]HPF, high-power field. [b]NS, not significant.

There are only few series on ILC that contain survival data from more than 100 patients, and follow-up of the patients in these series has been for fewer than 10 years except for a small proportion of patients. In line with the present findings, Dixon et al (1985) found in their series of 105 patients that those with ILC have better outcome than those with IDC. A similar conclusion was reached by Du Toit et al (1991) and Silverstein et al (1994) in their studies consisting of 171 and 161 patients respectively. DiCostanzo et al (1990) compared survival of 230 patients with ILC with stage-matched patients with IDC. Patients with stage I ILC were found to have significantly better survival than those with stage I IDC, but this difference disappeared when the comparison was carried out in stage II cancer. There was no difference in overall survival between ILC and other invasive carcinomas of the breast in the largest series of ILC published so far, consisting of 726 patients (Sastre-Garau et al, 1996), but follow-up in this series was relatively short with only 25 patients followed up for 10 years.

In the present series, ILCs were significantly more often of low histological grade, had lower mitotic counts, were less often necrotic and more often DNA diploid and had a lower SPF than IDCs. Furthermore, ILCs had axillary nodal metastases significantly less often than IDCs. The differences in the distribution of these important prognostic variables are compatible with the more favourable outcome of ILC compared with IDC. ILCs have also been found to be associated with a low histological grade by others (Silverstein et al, 1994; Sastre-Garau et al, 1996), but reports on the frequency of axillary nodal involvement are contradictory (Silverstein et al, 1994; Sastre-Garau et al, 1996). ILCs have been reported to express more often oestrogen and progesterone receptors (Helin et al, 1989; Sastre-Garau et al, 1996) and less often *erbB-2* and *p53* oncogenes (Toikkanen et al, 1992; Rosen et al, 1995) than IDCs, and their bcl-2 expression has been reported to be stronger than that of IDCs (Joensuu et al, 1994); these findings also suggest that ILC is biologically less aggressive than IDC.

Patients with node-negative ILC and with a primary tumour size 2 cm or less (pT1N0M0) had a 100% 10-year survival rate in our series, suggesting that small ILCs have as good a prognosis as tubular, mucinous, papillary and cribriform carcinomas, which are associated with a particularly favourable outcome. The finding that node-negative ILCs with a SPF less than 4% have a 100% 20-year survival rate also supports the view that there is a subgroup of ILC that can be defined by the current methodology and that is associated with excellent long-term survival even if no adjuvant therapy is given.

The presence of tumour necrosis was strongly associated with poor prognosis in the current series. To our knowledge, the value of tumour necrosis as a prognostic factor in ILC has not been previously investigated. ILCs with necrotic areas had only a 16% 10-year survival rate compared with 72% in ILC without necrosis, but the rarity of this feature (present only in < 10% of cases) limits its use as a prognostic factor.

We conclude that, in comparison to IDC:

1. ILC is associated with better short-term and long-term survival;
2. the distribution of several established prognostic factors is more favourable in ILC and they explain the better prognosis of ILC;
3. in addition to the primary tumour size and axillary nodal status, the presence of tumour necrosis and the cellular proliferation rate are important prognostic features in ILC;
4. contralateral breast cancer is more commonly diagnosed in patients with ILC;
5. the results suggest that there exist subgroups of ILC (pT1N0M0 or node-negative ILC with SPF ≤ 4%) that carry excellent long-term prognosis with the 20-year corrected survival rate approaching 100% even if treated without adjuvant therapy.

© Cancer Research Campaign 1997

### REFERENCES

Azzopardi JG (1983) In situ and invasive lobular carcinoma of the breast. In *New Frontiers in Mammary Pathology*, Hollman KH and Verley JM. (eds), pp. 127–145. Plenum Press: New York

Azzopardi JG, Ahmed A and Millis RR (eds) (1979) *Problems in Breast Pathology*. Vol. 11 in the series *Major Problems in Pathology*. WB Saunders: London

Correa P and Johnson WD (1978) International variation in the histology of breast carcinoma. *UICC Technical Report Series* **35**: 36–65

DiCostanzo D, Rosen PP, Gareen I, Franklin S and Lesser M (1990) Prognosis in infiltrating lobular carcinoma. An analysis of 'classical' and variant tumours. *Am J Surg Pathol* **14**: 12–23

Dixon JM, Anderson TJ, Page DL and Duffy SW (1982) Infiltrating lobular carcinoma of the breast. *Histopathology* **6**: 149–161

du Toit RS, Locker AP, Ellis IO, Elston CW, Nicholson RI, Robertson JF and Blamey RW (1991) An evaluation of differences in prognosis, recurrence patterns and receptor status between invasive lobular and other invasive carcinomas of the breast. *Eur J Surg Oncol* **17**: 251–257

Eusebi V, Betts C, Haagensen DE, Gugliotta P, Bussolati G and Azzopardi JG (1984) Apocrine differentiation in lobular carcinoma of the breast: a morphologic, immunologic, and ultrastructural study. *Hum Pathol* **15**: 134–140

Fechner RE (1972) Infiltrating lobular carcinoma without lobular carcinoma in situ. *Cancer* **29**: 1539–1546

Fechner R (1975) Histologic variants of infiltrating lobular carcinoma of the breast. *Hum Pathol* **6**: 373–378

Fisher ER, Gregorio RM, Fisher B, Redmond C, Vellios F and Sommers SC (1975) The pathology of invasive breast cancer. A syllabus derived from findings of the National Surgical Adjuvant Breast Project (protocol no. 4). *Cancer* **36**: 1–85

Fisher ER, Gregorio RM, Redmond C and Fisher B (1977) Tubulolobular invasive breast cancer: a variant of lobular invasive cancer. *Hum Pathol* **8**: 679–683

Foote FW Jr and Stewart FW (1941) Lobular carcinoma in situ: a rare form of mammary cancer. *Am J Pathol* **17**: 491–496

Foote FW Jr and Stewart FW (1946) A histological classification of carcinoma of the breast. *Surgery* **19**: 74–99

Helin HJ, Helle MJ, Kallioniemi O-P and Isola JJ (1989) Immunohistochemical determination of estrogen and progesterone receptors in human breast carcinoma. Correlation with histopathology and DNA flow cytometry. *Cancer* **63**: 1761–1767

Howell A and Harris M (1985) Infiltrating lobular carcinoma of the breast. *Br Med J* **291**: 1371–1372

Joensuu H and Toikkanen S (1995) Cured of breast cancer? *J Clin Oncol* **13**: 62–69

Joensuu H, Pylkkänen L and Toikkanen S (1994) Bcl-2 protein expression and long-term survival in breast cancer. *Am J Pathol* **145**: 1191–1198

Martinez VM and Azzopardi JG (1979) Invasive lobular carcinoma of the breast: incidence and variants. *Histopathology* **3**: 467–488

Newman W (1966) Lobular carcinoma of the female breast. Report of 73 cases. *Ann Surg* **164**: 305–314

Rosen PP, Lesser ML, Arroyo CD, Cranor M, Borgen P and Norton L (1995) p53 in node negative breast carcinoma: an immunohistochemical study of epidemiologic risk factors, histologic features, and prognosis. *J Clin Oncol* **13**: 821–830

Sastre-Garau X, Jouve M, Asselain B, Vincent-Salomon A, Beuzeboc P, Dorval T, Durand J-C, Fourquet A and Pouillart P (1996) Infiltrating lobular carcinoma of the breast: clinicopathologic analysis of 975 cases with reference to data on conservative therapy and metastatic spread. *Cancer* **77**: 113–120

Silverstein MJ, Lewinsky BS, Waisman JR, Gierson ED, Colburn WJ, Senofsky GM and Gamagmi P (1994) Infiltrating lobular carcinoma. Is it different from infiltrating duct carcinoma? *Cancer* **73**: 1673–1677

Steinbrecher JS and Silverberg SG (1976) Signet-ring cell carcinoma of the breast. The mucinous variant of infiltrating lobular carcinoma? *Cancer* **37**: 828–840

Toikkanen S, Joensuu H and Klemi P (1989) The prognostic significance of nuclear DNA content in invasive breast cancer – a study with long-term follow-up. *Br J Cancer* **60**: 693–700

Toikkanen S, Helin H, Isola J and Joensuu H (1992) Prognostic significance of HER-2 oncoprotein expression in breast cancer: a 30-year follow-up. *J Clin Oncol* **10**: 1044–1048

UICC (International Union against Cancer) (1987) *TNM classification of malignant tumours* (4th revised edn). Springer Verlag: Berlin

World Health Organization (1981) *Histological Typing of Breast Tumours*. International Histological Classification of Tumours, No. 2 (2nd edn). World Health Organization: Geneva

*© Cancer Research Campaign 1997*